# Impact of tele-ultrasound on novice users in patients with suspected COVID-19 in an urgent care setting

**DOI:** 10.3389/fdgth.2025.1703121

**Published:** 2026-01-21

**Authors:** John L. Kendall, Sarah Janik, Paul Khalil, Tim Scheel, Michael Breyer, Stacy A. Trent, Matthew Riscinti

**Affiliations:** 1 Department of Emergency Medicine, Stanford University School of Medicine, Stanford, CA, United States; 2 Department of Emergency Medicine, Denver Health Medical Center, Denver, CO, United States

**Keywords:** advanced practice clinician, COVID-19, lung ultrasonography, POCUS, tele-ultrasound

## Abstract

**Background:**

Point-of-care lung ultrasound (LUS) has been described for the evaluation of lung pathologies such as pneumothorax, pneumonia, and COVID-19 infections. It is rapidly deployed, portable, and accurate for LUS diagnoses. However, a learning curve limits its use, and teleguidance has been proposed as a solution. In this study, we primarily seek to measure the effect of tele-guided lung ultrasound (T-LUS) on chest X-ray (CXR) utilization in patients presenting with COVID-19 symptoms. Secondarily, we measure the effect of T-LUS on clinical decision-making, length of stay, and clinical outcomes.

**Results:**

We performed a retrospective observational study using a before–after design in an adult urgent care (AUC) setting. A total of 303 patients with symptoms suggestive of COVID-19 were included. AUC providers used T-LUS on 31% of patients with COVID-19 symptoms (*n* = 34). Abnormal LUS findings were found in 41% of patients (*n* = 14), with B-lines (86%) and pleural irregularities (79%) being the most common findings. Among all patients in the study period, those who received a T-LUS did not show a statistically significant difference in CXR utilization [−12% difference; 95% confidence interval (CI) −25% to 5%] as compared to patients who did not receive a T-LUS, and a similarly non-significant difference was observed in the intervention period (−5% difference; 95% CI: −21% to 14%). Length of stay was longer for patients in whom T-LUS was used (median difference 26 min, 95% CI 11–41). However, a comparison of patients in the intervention period revealed no significant difference in length of stay between patients who received T-LUS and those that did not (median difference 16 min, 95% CI −5 to 37).

**Conclusion:**

T-LUS is feasible and alters clinical decision-making for novice ultrasound users in the care of patients with suspected COVID-19 infection. Our results indicated that there was a no statistically significant difference trend in CXR utilization and no improvement in length of stay by the end of the 2-week trial.

## Introduction

Several imaging modalities have been used to evaluate patients with respiratory symptoms related to suspected COVID-19 infections, including chest X-ray (CXR), chest computed tomography (CT), and lung ultrasound (LUS). Chest X-ray and CT, however, have limitations such as poor specificity for COVID-19, radiation exposure to patients, potential increased risk of COVID-19 exposure to healthcare personnel, and increased cleaning and decontamination time for equipment (1). In the early stages of the pandemic, many patients suspected of having COVID-19 infection received a CXR as part of their evaluation, yet the findings in many were unremarkable and likely did not meaningfully contribute to patient management decisions. Lung ultrasound is a portable, non-invasive, rapid diagnostic technique capable of detecting low-risk COVID-19 patients (2), with the potential to reduce exposure of hospital personnel and equipment to pathogens (3, 4). Specifically, a normal LUS examination has been utilized by medical care providers to reduce the utilization of CXR in low-risk patients (5).

Many patients with infectious respiratory symptoms present to urgent and primary care acute care centers. Commonly, these settings have limited access to point-of-care ultrasound (POCUS), and providers often lack ultrasound training to perform LUS. Therefore, ultrasound is infrequently used in the initial evaluation of patients with suspected COVID-19 infections. Tele-ultrasound (TUS) is defined as the transmission of ultrasound images from one location to another for interpretation (6). When it occurs synchronously, feedback can be provided to non-expert sonographers to guide image acquisition and interpretation. Research has been conducted in a number of different environments, with a variety of care providers demonstrating that TUS is feasible (7). Despite this, very few studies have addressed the impact of TUS programs on the utilization of resources, length of stay, clinical decision-making, and patient outcomes.

In this study, we developed a TUS program in an urgent care setting for advanced practice providers (APPs) to perform LUS on patients with respiratory complaints and suspected COVID-19 infection. Given the practice that was followed in the early stages of the pandemic, wherein many patients received a portable CXR as part of their evaluation, and the evidence that LUS can safely reduce CXR utilization in low-risk patients, we identified CXR utilization as our primary outcome in this study. Our primary aim was to measure the effect of T-LUS on CXR utilization. Secondarily, we sought to determine the effect of T-LUS on clinical decision-making, patient length of stay, and clinical outcomes.

## Materials and methods

### Setting and population

This study was performed in the adult urgent care (AUC) at Denver Health Medical Center, a regional, academic, level-1 trauma center and safety-net hospital in Denver, Colorado. The AUC is a 23-bed facility that attends to patients 7 days a week (13 h on weekdays and 11 h on weekends). Patients are seen primarily by APPs (e.g., physician assistants or nurse practitioners) working alongside a single, board-certified emergency medicine physician.

The AUC is colocated with the adult emergency department (ED), and both utilize the same centralized, nurse-driven, triage process. At triage, ED nursing staff obtain basic details from patients such as the patient's chief complaint, age, heart rate, and oxygen saturation levels. To be eligible for triage to the AUC, patients must not be ill-appearing, unstable (oxygen saturation <90% or heart rate >130), altered, intoxicated, having suicidal tendencies, non-ambulatory, or complaining of acute neurologic deficits or trunk trauma.

### Context

At the onset of the COVID-19 pandemic, adult patients presenting with symptoms concerning for COVID-19 were triaged to a specific zone in the adult ED regardless of their age, symptoms, or vital signs in order to minimize exposure to staff and preserve personal protective equipment. If an ED provider ordered a CXR, it was obtained at the bedside as a single anterior–posterior view to minimize the transport of potentially infectious patients through other zones of the ED and also minimize exposure to radiology staff. In the first weeks of the COVID-19 pandemic, APPs had limited to no exposure to COVID-19 patients, given our cohorting strategy. However, as the AUC number of patient encounters rapidly fell and the adult ED number of patient encounters climbed, we reinstated our pre-COVID triage protocols to utilize more physical space in the AUC to attend to stable patients with symptoms concerning for COVID-19.

The impetus for this pilot project stemmed from the desire to minimize unnecessary CXRs that were unlikely to change patient management.

### Design and implementation of intervention

For this pilot intervention, a Butterfly iQ + handheld ultrasound transducer (Butterfly Network, Inc., Guilford, CT, USA) and iPad mini were stored in the AUC and readily accessible to APPs. At the outset of the study, an email was sent to all APPs describing the study, defining the entry criteria, and providing the contact number of the on-call physician. A flyer with the same information was posted in the APP workspace in the AUC. When an eligible patient was identified, the APP would text an on-call remote POCUS expert. These experts consisted of three ultrasound-trained emergency medicine faculty members and three current ultrasound fellows. Each remote ultrasound expert had extensive experience with LUS and had participated in the development of standard nomenclature and characterization of the different pathologic lung findings of COVID-19 infection.

**Figure 1:**
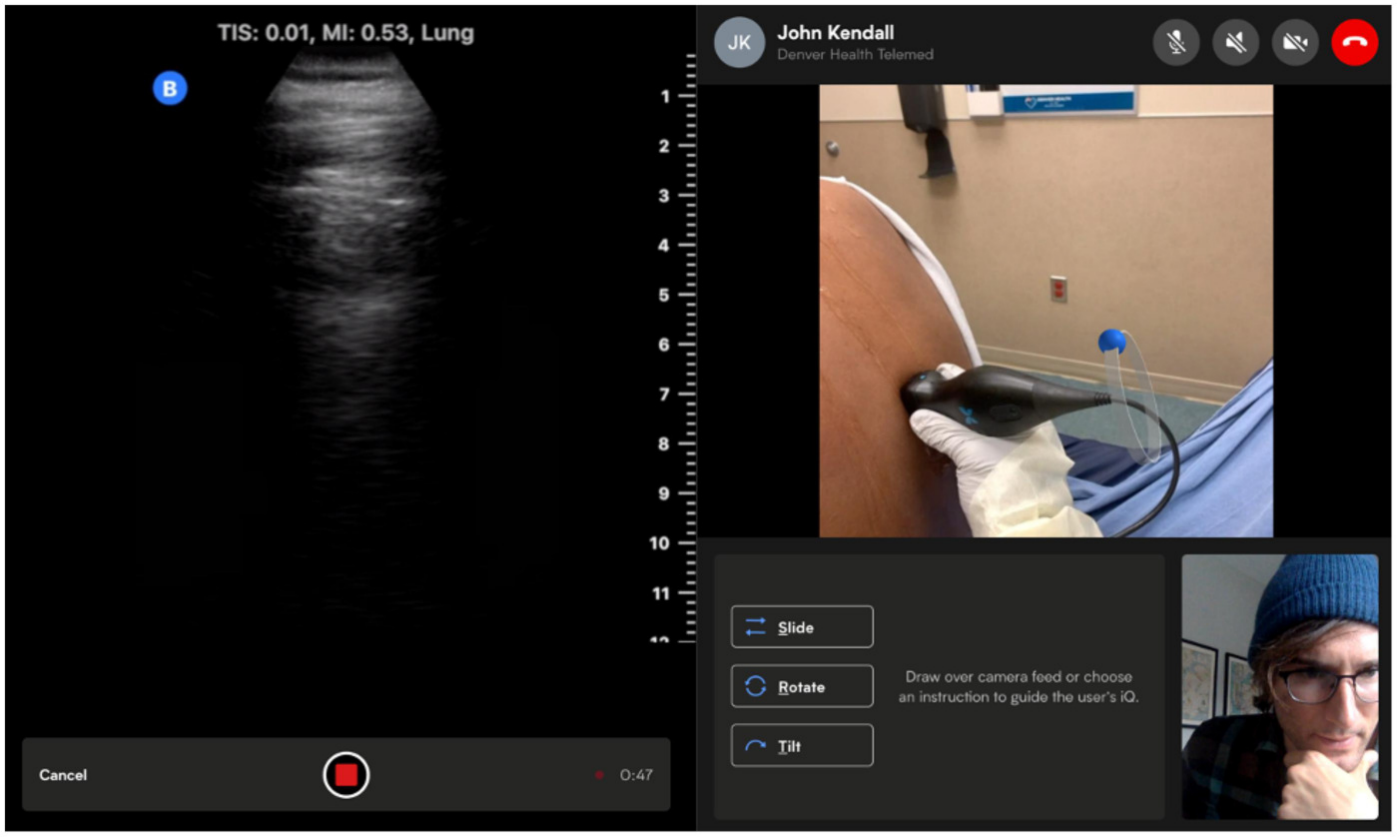
A tele-ultrasound user interface. Figure contains images of the author(s) only.

Remote TUS sessions were initiated by the APP in the Butterfly iQ + teleguidance software interface (6). Once established, the TUS interface provided the remote POCUS expert with an audio link and real-time images, supplemented by simultaneous images of the scanner's hand, to allow spatial orientation (Figure 1).

Imaging parameters were controllable by the remote expert (e.g., preset, depth, gain, and Doppler) who had access to the functionality of screen annotations to help direct probe positioning and movement by the APP. The remote POCUS expert was also able to guide the APP with verbal instructions. Each scan included views of the upper and lower lung zones anteriorly and posteriorly on each hemithorax for a total of eight zones evaluated. Each view was recorded by acquiring a short video segment that was uploaded to HIPAA-compliant cloud storage through the Butterfly software interface.

### Study of the intervention

We conducted a retrospective observational study using a before–after design. This study was approved by our institution's quality improvement review committee. In addition, this study is reported in accordance with the Standards for Quality Improvement Reporting Excellence (SQUIRE 2.0) guidelines (8).

The study cohort consisted of a 2-week preintervention group (23 March 2020, through 5 April 2020) and a 2-week postintervention group (6 April 2020, through 23 April 2020). We assembled a base cohort electronically using Microsoft SQL Server Management Studio to query the Epic Cogito Data Warehouse at Denver Health Medical Center for all patients seen by an AUC provider during this period. Patient demographics (e.g., age, sex, and race/ethnicity), primary insurance, triage acuity level, name of the APP, CXR obtained (yes/no), any advanced imaging (CT, MRI, or radiology-performed ultrasound) obtained, throughput dates and times, primary urgent care diagnosis, and disposition were electronically extracted from Epic for all AUC patients seen during this period. All patients were stratified into two groups based on a suspicion of COVID-19, which was identified by any ICD-10 code specific to COVID-19 or a primary diagnosis suggestive of COVID-19, which broadly included cough, respiratory illness/infection, or viral syndrome/illness. At the time of this study, COVID-19 testing was not being done on patients who were not being admitted to the hospital.

For patients in whom the T-LUS program was used, additional data aimed at measuring the effect of T-LUS on clinical decision-making were collected by the POCUS expert prior to obtaining ultrasound images. This included the APP's suspicion of COVID-19 (low/high), prior use of the T-LUS program (yes/no), and whether the APP initially planned to obtain CXR (yes/no). The question on whether a CXR was planned was asked prospectively before any imaging order was placed and prior to the T-LUS session. The time to complete the T-LUS image capture and interpretation was recorded in seconds by the TUS software. The timer was started when the ultrasound probe was initially placed on the skin and stopped when the scanning session was completed. After ultrasound images were obtained, the POCUS expert also documented the APP's “ease of use” of the T-LUS program, which was categorized as “easy,” “moderate,” and “hard.” The POCUS expert also documented their interpretation of the LUS images including the presence or absence of B-lines, pleural irregularities, and subpleural consolidations. An LUS without any of these findings was considered “normal.” Lastly, for all patients in whom the T-LUS program was used, we retrospectively reviewed the medical record to ascertain whether the patient had any heart or lung diseases that could make the LUS abnormal at baseline (i.e., chronic obstructive pulmonary disease, asthma, or congestive heart failure). We also reviewed the medical record to identify the results of the COVID-19 test obtained prior to the encounter and for 2 weeks following the intervention period to determine whether a return visit occurred and whether there was alleviation or worsening of symptoms.

Provider to disposition time was defined as the time in minutes from when an APP assigns themselves to a patient to the time the APP assigns a patient disposition. Patient length of stay was defined as the time in minutes from patient arrival to the time the patient physically leaves the AUC.

### Data management and statistical analyses

Data for all AUC patients during the study period were electronically captured from Epic and exported into an electronic spreadsheet (Microsoft Excel, Redmond, WA, USA) and subsequently transferred into SAS Enterprise Guide 7.0 (SAS Institute, Inc., Cary, NC, USA). Additional data for all AUC patients who utilized the T-LUS program were manually entered into an electronic spreadsheet (Microsoft Excel), transferred into SAS Enterprise Guide, and subsequently merged with the Epic data. Descriptive statistics were performed for all variables. Prevalence estimates with 95% confidence intervals (CIs) were used to report the prevalence of CXR use. Estimates of effect size (percent or median difference) with 95% CIs were made to test the effect of the intervention on CXR use, provider to disposition, and patient length of stay. A repeated measures model using SAS PROC MIXED was prepared to determine the effect of repeated use of the T-LUS program per APP on time to complete T-LUS.

## Results

Table 1 describes the patient and urgent care characteristics during the study period.

**Table 1:** Patient and urgent care characteristics.

Patient and urgent care characteristics	All patients (*N* = 1,407)	Precohort		Postcohort	
		All patients (*n* = 682)	COVID symptoms (*n* = 189)	All patients (*n* = 725)	COVID symptoms (*n* = 114)
Age^a^	36 (28–48)	36 (29–48)	35 (29–48)	36 (28–48)	33 (27–44)
Female sex, *n* (%)	659 (47)	296 (43)	88 (47)	363 (50)	49 (43)
Race/ethnicity, *n* (%)					
Hispanic	608 (43)	307 (45)	83 (44)	301 (42)	57 (50)
Non-Hispanic White	508 (36)	232 (34)	60 (32)	276 (38)	30 (26)
Non-Hispanic Black	211 (15)	99 (15)	26 (14)	112 (15)	18 (16)
Other	80 (6)	44 (6)	20 (11)	36 (5)	9 (8)
Primary insurance, *n* (%)					
Medicaid	695 (49)	323 (47)	82 (43)	372 (51)	43 (38)
Uninsured	312 (22)	182 (27)	54 (29)	130 (18)	23 (20)
Private	279 (20)	119 (17)	44 (23)	160 (22)	44 (39)
Medicare	112 (8)	55 (8)	8 (4)	57 (8)	4 (4)
Other	9 (1)	3 (0)	1 (0)	6 (1)	0
ESI level, *n* (%)					
2	2 (0)	2 (0)	1 (0)	0	0
3	549 (39)	246 (36)	60 (32)	303 (42)	48 (42)
4	685 (49)	334 (49)	83 (44)	351 (48)	61 (54)
5	171 (12)	100 (15)	43 (23)	71 (10)	4 (4)
Imaging obtained, *n* (%)					
Chest X-ray	186 (13)	114 (17)	80 (42)	72 (10)	34 (30)
Any X-ray	392 (28)	209 (31)	80 (42)	183 (25)	35 (31)
Advanced imaging	244 (17)	99 (15)	3 (2)	145 (20)	4 (4)
Throughput^a^					
Daily census	51 (40–60)	50 (39–58)	15 (9–17)	51 (42–61)	7 (5–11)
Provider to disposition	72 (39–124)	65 (34–115)	55 (27–85)	78 (44–137)	73 (41–112)
Length of stay	99 (64–155)	90 (59–142)	78 (52–115)	108 (70–166)	96 (68–133)
Disposition, *n* (%)					
Discharge	1,338 (95)	652 (96)	18 (98)	687 (95)	112 (98)
Admit	68 (5)	30 (4)	54 (2)	38 (5)	2 (2)

ESI, emergency severity index.

a Median minutes (interquartile range).

A total of 1,407 patients were examined in the AUC over the course of the study period, including 303 patients with diagnoses concerning for COVID-19. The median age of the patients was 36 [interquartile range (IQR) 28–48]; 47% were female; Hispanics were the largest race/ethnicity group (43%). Radiographs were obtained in 28% of all AUC patients, including 42% of patients with COVID symptoms prior to the T-LUS intervention and 30% of patients with COVID symptoms during the T-LUS intervention. None of the patients in the T-LUS intervention group had a positive COVID-19 test prior to enrollment and none had comorbidities that could have affected the LUS findings. The median patient length of stay was 99 min (IQR 64–155) for all patients during the study period.

Table 2 describes the T-LUS program use and findings.

**Table 2:** Tele-ultrasound characteristics.

Tele-ultrasound charactertics	*n* (%)
Frequency of use in patients w/COVID symptoms	34 (31)
APPs’ prior use of T-LUS in COVID patients	
0%	8 (40)
1%–25%	2 (10)
26%–50%	7 (35)
>50%	3 (15)
APPs reported ease of use	
Easy	30 (88)
Moderate	4 (12)
Hard	0
APPs planned CXR prior to T-LUS	19 (56)
Abnormal T-LUS readings	14 (41)
Abnormal T-LUS findings	
B-lines present	12 (86)
Pleural irregularities	11 (79)
Subpleural consolidation	3 (21)
Time to complete T-LUS, median (IQR)	8 (6–10)

**Figure 2:**
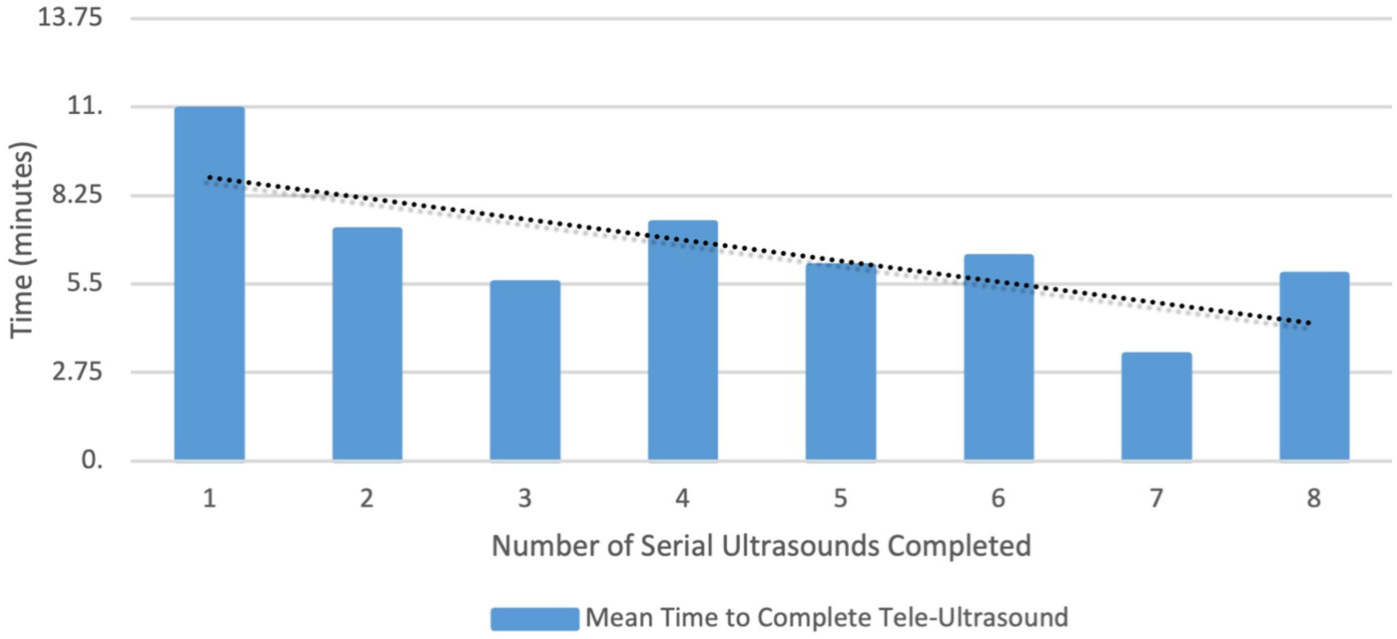
The mean time to complete tele-ultrasound controlling for repeated measures by the provider.

The APPs used T-LUS on 31% of patients with COVID-19 symptoms (*n* = 34), and 60% of APPs utilized T-LUS on at least one of their patients with COVID-19 symptoms. Abnormal LUS findings were found in 41% of patients (*n* = 14), with B-lines (86%) and pleural irregularities (79%) being the most common findings. The median time to complete the T-LUS was 8 min (IQR 6–10). Figure 2 shows the effect of repeated use of T-LUS by the same APP on time to complete a T-LUS. The mean time to complete T-LUS significantly decreased with repeated use (*p* = 0.02).

Tables 3, 4 describe the effect of T-LUS on clinical outcomes.

**Table 3:** Effect of T-LUS on CXR use and throughput in all patients with COVID symptoms.

Outcome measure	All COVID symptoms (*n* = 303)	T-LUS (*n* = 34)	No T-LUS (*n* = 269)	Percentile or median difference (95% CI)
CXR use, *n* (%)	113 (37)	9 (26)	104 (39)	−12 (−25 to 5)
Provider to disposition, median (IQR)	61 (31–94)	80 (59–117)	58 (29–90)	20 (5–34)
Length of stay, median (IQR)	88 (57–122)	113 (91–144)	82 (55–119)	26 (11–41)

**Table 4:** Effect of T-LUS on CXR use and throughput in patients with COVID symptoms in the intervention period.

Outcome measure	All COVID symptoms (*n* = 114)	T-LUS (*n* = 34)	No T-LUS (*n* = 80)	Percentile or median difference (95% CI)
CXR use, *n* (%)	32 (29)	9 (26)	23 (31)	−5 (−21 to 14)
Provider to disposition, median (IQR)	73 (41–112)	80 (59–117)	62 (34–108)	16 (−6 to 38)
Length of stay, median (IQR)	96 (68–133)	113 (91–144)	92 (62–132)	16 (−5 to 37)

Over the 4-week period, CXR was obtained in 37% of patients with diagnoses suggestive of COVID-19. Among all patients in the study period, those who received a T-LUS did not show a statistically significant difference in CXR utilization (−12% difference; 95% CI −25% to 5%) as compared to those who did not receive a T-LUS, and a similarly non-significant difference was observed in the intervention period (−5% difference; 95% CI: −21% to 14%). Across the study period, length of stay was significantly longer for patients in whom T-LUS was used (median difference 26 min, 95% CI 11–41). While not statistically significant, this trend was also observed in the intervention period (median difference 16 min, 95% CI −5 to 37).

The decision to obtain a CXR was changed in 15 of the 34 patients (44%) who received a T-LUS. In 11 patients with normal LUS findings, the APPs did not order a CXR when they had planned to order one. Conversely, in four patients with abnormal LUS findings, the APPs did order a CXR when they had planned to not order one. Of the 34 patients, there were 10 patients who had either phone or in-person follow-up. Eight reported alleviation in their symptoms. Of the two patients with worsening symptoms, one had a repeat CXR obtained, which was read as “no acute cardiopulmonary abnormalities,” and the second patient only complained about a sore throat, and therefore, a CXR was not obtained. The remaining 24 patients did not have a return visit within the 2-week period after they were discharged.

## Discussion

Tele-ultrasound is a specific use of telemedicine where ultrasound images are transmitted to facilitate remote image acquisition and/or interpretation. Prior studies have addressed the feasibility of TUS technology, but very few have reported on the impact of outcomes such as resource utilization or decision-making (6). In our pilot study, we found that T-LUS was feasible and could alter clinical decision-making for novice ultrasound users in the care of patients with suspected COVID-19 infection. Feasibility in this pilot was determined by the successful completion of a T-LUS study, the ability to acquire interpretable images, the high ease-of-use ratings (88%), and the short median time to complete an examination (8 min).

The majority of TUS programs are reported in low-resource settings, specialty clinics, or austere environments (6, 7). Ours is the first to present results from implementation in an AUC setting with APPs. We found that T-LUS was performed in 31% of patients presenting with suspected COVID-19 infection with a median time to complete the examination of 8 min. In addition, the APPs rated the T-LUS examination as “easy” to perform in 88% of the patients. Barriers to implementing TUS programs include a platform to send images to an expert and a communication system. In many instances, cost of equipment is an additional barrier. In this study, we deployed a hardware and software solution developed by Butterfly Network, Inc. The interface allows remote experts to visualize transducer position and ultrasound images in real time with the ability to control ultrasound settings, including depth, gain, color Doppler, and image capture. The remote POCUS expert can also provide visual commands to the sonographer for probe positioning and movement in addition to being able to verbally guide image acquisition and interpretation. This platform has been previously described and utilized in military deployed environments and to facilitate remote ultrasound-guided vascular access (4, 9).

A secondary aim of our study was to observe the effect of a T-LUS program on CXR utilization in patients presenting to an AUC with suspected COVID-19 infections. While the point estimate of CXR utilization declined by 12% over the study period, it was not statistically significant. Moreover, we observed less difference in CXR utilization in the intervention period (5%), suggesting that the observed trend in CXR utilization over the study period may be more reflective of a secular trend toward decreased CXR utilization in this stable urgent care population, given the increased comfort by providers on how to manage COVID-19. We did, however, observe that T-LUS altered decision-making around CXR utilization for individual patients in both directions. Normal T-LUS findings resulted in our not obtaining a CXR that was previously planned, and abnormal T-LUS findings resulted in our obtaining a CXR that was not previously planned. This suggests increased accuracy of LUS in COVID-19 patients, which has also been described previously (2).

While length of stay did not significantly increase during the intervention period, we did observe a modest increase across the study period and a trend toward an increase in the intervention period when T-LUS was used. While the median time to complete a T-LUS session was only 8 min, there was additional time to locate and set up the equipment, contact the remote expert, and complete the paperwork that was not included in the timing recorded for the T-LUS session. Consequently, a small increase in length of stay with the use of T-LUS is not surprising. Interestingly, there appeared to be a learning curve that resulted in a significantly decreased time to complete a T-LUS session with APPs who had performed more than seven studies (Figure 2).

The results of our study can likely be extrapolated to many different clinical care areas. While we studied an AUC setting with APPs, it is conceivable that a similar program with similar results can be used in other settings with novice sonographers, such as primary care clinics, nursing homes, or home health settings.

Our study had a number of limitations that may have influenced our findings. First, our sample size was small, as also the percentage of patients with suspected COVID-19 who received a T-LUS examination. Given this limited sample size, the analysis may have been underpowered to detect modest differences between groups, and it is plausible that we would have obtained different results with a greater use of T-LUS. In addition, since an AUC setting generally handles a lower acuity patient population, it can be argued that there is limited utility in obtaining a CXR, regardless of the ultrasound findings. While this may be true, we initiated this study when we recognized that close to 40% of patients with suspected COVID-19 infection had a CXR performed on them. Subsequent studies will need to determine whether CXR utilization is maintained in low-risk patients with suspected COVID-19 as care providers become more familiar with the diagnosis and management.

Second, our before–after observational study design introduces important limitations. There may have been significant differences in the acuity of the patients between groups. Also, clinical practice patterns related to the COVID-19 diagnosis and management evolved substantially over the study period, and these changes may have influenced CXR utilization, independent of T-LUS utilization. We collected only Emergency Severity Index information from the cohorts, and therefore, robust comparisons are not possible. Other factors such as volume of patients and provider staffing may also have been different between cohorts.

Operational considerations may have also played a role. We did not measure adherence to the T-LUS workflow or to other factors such as APP workload, competing clinical demands, or extended lengths of stay, which may have influenced the consistent use of T-LUS. There may have been differences in T-LUS use depending on whether an on-call POCUS expert was available. Lastly, the TUS platform and the guidance features that we deployed are specific to the Butterfly iQ + ultrasound system, which limits generalizability.

It can be concluded that tele-lung ultrasound can be successfully implemented in an AUC setting with novice sonographers and it can alter clinical decision-making in patients with suspected COVID-19 infection. In this study, we found that there was no statistically significant reduction in CXR utilization, and length of stay remained unchanged in the T-LUS cohort. Time to complete a T-LUS session decreased significantly with greater experience with the platform.

## Data Availability

The raw data supporting the conclusions of this article will be made available by the authors without undue reservation.
